# Supplementary material for: From sequence to enzyme mechanism using multi-label machine learning
Source: BMC Bioinformatics. 2014 May 19;15:150. doi: 10.1186/1471-2105-15-150 (PMC4229970; doi:10.1186/1471-2105-15-150)
Supplement: Additional file 2 — Java code of ml2db. Additional file ml2db_code.tar.gz contains the Java source code to run the multi-label machine learning experiments and save the results to database. The code’s Javadoc is included. [file 1471-2105-15-150-S2.zip › additional file 2/ml2db/ml2db/doc/mulan/evaluation/measure/MacroAUC.html]

MacroAUC


JavaScript is disabled on your browser.


- Overview
- Package
- Class
- Use
- Tree
- Deprecated
- Index
- Help

- Prev Class
- Next Class

- Frames
- No Frames

- All Classes

- Summary:
- Nested |
- Field |
- Constr |
- Method

- Detail:
- Field |
- Constr |
- Method


mulan.evaluation.measure

## Class MacroAUC

- java.lang.Object
- - mulan.evaluation.measure.MeasureBase
  - - mulan.evaluation.measure.ConfidenceMeasureBase
    - - mulan.evaluation.measure.LabelBasedAUC
      - - mulan.evaluation.measure.MacroAUC

- All Implemented Interfaces:
  :   java.io.Serializable, MacroAverageMeasure, Measure

  ---

    

  ```
  public class MacroAUC
  extends LabelBasedAUC
  implements MacroAverageMeasure
  ```

  Implementation of the macro-averaged AUC measure.

  Version:
  :   2010.12.10

  Author:
  :   Grigorios Tsoumakas

  See Also:
  :   Serialized Form

- - ### Constructor Summary

    Constructors

    Constructor and Description

    `MacroAUC(int numOfLabels)`

    Creates a new instance of this class
  - ### Method Summary

    Methods

    | Modifier and Type | Method and Description |
    | --- | --- |
    | `java.lang.String` | `getName()` Gets the name of a measure. |
    | `double` | `getValue()` Gets the value of a measure. |
    | `double` | `getValue(int labelIndex)` Returns the AUC for a particular label |

    - ### Methods inherited from class mulan.evaluation.measure.LabelBasedAUC

      `getIdealValue, reset`
    - ### Methods inherited from class mulan.evaluation.measure.MeasureBase

      `makeCopy, toString, update`
    - ### Methods inherited from class java.lang.Object

      `equals, getClass, hashCode, notify, notifyAll, wait, wait, wait`

- - ### Constructor Detail


    - #### MacroAUC

      ```
      public MacroAUC(int numOfLabels)
      ```

      Creates a new instance of this class

      Parameters:
      :   `numOfLabels` - the number of labels
  - ### Method Detail


    - #### getName

      ```
      public java.lang.String getName()
      ```

      **Description copied from interface: `Measure`**

      Gets the name of a measure.

      **Specified by:**
      :   `getName` in interface `Measure`

      Returns:
      :   the name of a measure.


    - #### getValue

      ```
      public double getValue()
      ```

      **Description copied from interface: `Measure`**

      Gets the value of a measure. The measure is incrementally cumulated for learner's
      prediction by each `Measure.update(MultiLabelOutput, boolean[])` call. The value
      returned by the method, returns sum of all update calls divided by the number
      of calls (average of all measures for all predictions).

      **Specified by:**
      :   `getValue` in interface `Measure`

      Returns:
      :   the average measure value computed so far


    - #### getValue

      ```
      public double getValue(int labelIndex)
      ```

      Returns the AUC for a particular label

      **Specified by:**
      :   `getValue` in interface `MacroAverageMeasure`

      Parameters:
      :   `labelIndex` - the index of the label

      Returns:
      :   the AUC for that label


- Overview
- Package
- Class
- Use
- Tree
- Deprecated
- Index
- Help

- Prev Class
- Next Class

- Frames
- No Frames

- All Classes

- Summary:
- Nested |
- Field |
- Constr |
- Method

- Detail:
- Field |
- Constr |
- Method
